# A single postoperative red cell distribution width measurement predicts short- and long-term mortality in surgical patients

**DOI:** 10.1007/s10354-026-01129-8

**Published:** 2026-01-25

**Authors:** Daniel Dankl, Bernhard Wernly, Niklas Rodemund, Richard Rezar, Barbara Schreiber, Andreas Koköfer

**Affiliations:** 1 Clinic of Anaesthesiology, Perioperative Medicine and Intensive Care Medicine, Paracelsus Medical University Salzburg, Salzburg, Austria; 2 Center for Public Health and Healthcare Research, Paracelsus Medical University Salzburg, Salzburg, Austria; 3 Paracelsus Medical University Salzburg, Department of Internal Medicine 1, Salzburg, Austria; 4 Clinic of Internal Medicine II, Department of Cardiology, Paracelsus Medical University of Salzburg, Salzburg, Austria

**Keywords:** RDW, Risk stratification, ICU prognosis, Acute kidney injury, RDW, Risikostratifizierung, Prognose von ICU Patienten, Akutes Nierenversagen

## Abstract

**Background:**

The red cell distribution width (RDW) measures erythrocyte size variability and is linked to increased mortality in various diseases, including cardiovascular, kidney, and liver conditions. In critically ill patients, particularly those with sepsis, RDW is a prognostic marker. While its role in cardiac surgery patients is well established, its value in surgical patients is less well explored. This study investigates whether a single postoperative RDW measurement predicts short- and long-term mortality surgical intensive care unit (ICU) patients.

**Methods:**

This retrospective cohort study analyzed data from 2312 surgery patients admitted to a surgical ICU over 2 years. The RDW was measured within 2 h of surgery and analyzed as a continuous and binary variable (threshold 15). Mortality at 30 days and 1 year was assessed using univariable and multivariable logistic regression and Cox regression models, adjusting for factors like the simplified acute physiology score 3 (SAPS3), lactate levels, age, sex, and comorbidities. Interaction analyses evaluated the impact of cofactor on RDW’s mortality prediction.

**Results:**

Of 2312 patients, 1687 (73.0%) had RDW < 15 and 625 (27.0%) had RDW ≥ 15. The RDW ≥ 15 patients were older (*p* < 0.001), had higher SAPS3 (*p* < 0.001), and more comorbidities. Elevated RDW was independently associated with increased 30-day and 1‑year mortality. In univariable analysis, each unit increase in RDW was linked to higher 30-day mortality (HR 1.169, 95% CI 1.110–1.230; *p* < 0.001) and 1‑year mortality (HR 1.153, 95% CI 1.122–1.186; *p* < 0.001). Moreover, RDW ≥ 15 significantly increased the risk of 30-day (HR [Hazard Ratio] 3.247, 95% CI 2.352–4.482; *p* < 0.001) and 1‑year mortality (HR 3.278, 95% CI 2.654–4.048; *p* < 0.001). Interaction analyses showed that lactate levels and pre-existing lung diseases influenced RDW’s mortality prediction. Including RDW in multivariable models improved predictive accuracy, as indicated by the Akaike information criterion.

**Conclusion:**

Postoperative RDW is a reliable and cost-effective marker for predicting short- and long-term mortality in surgical patients. An RDW threshold of 15 identifies high-risk patients who may benefit from targeted follow-up, thus potentially improving outcomes. In summary, RDW enhances postoperative risk stratification and management.

## Abstract (de)

**Hintergrund:**

Die Erythrozytenverteilungsbreite (RDW) misst die Variabilität der Erythrozytengröße und ist mit erhöhter Mortalität bei verschiedenen Erkrankungen verknüpft. Bei kritisch kranken Patient:innen, besonders bei Sepsis, ist die RDW ein Prognosemarker. Während die Rolle der RDW bei herzchirurgischen Patient:innen gut belegt ist, wurde die prognostische Wertigkeit bei nicht herzchirurgischen Patient:innen weniger erforscht. In der vorliegenden Studie wurde untersucht, ob eine einzelne postoperative RDW-Bestimmung ein Prädiktor für Kurz- und Langzeitmortalität bei chirurgischen Intensivpatient:innen ist.

**Methoden:**

Dazu wurden in der vorliegenden retrospektiven Kohortenstudie Daten von 2312 chirurgischen Patient:innen analysiert, die im Verlauf von 2 Jahren auf einer chirurgischen Intensivstation aufgenommen wurden. Die RDW wurde innerhalb von 2 h nach der Operation gemessen und sowohl als kontinuierliche als auch als binäre Variable (Grenzwert 15) analysiert. Die Mortalität nach 30 Tagen und nach einem Jahr wurde mittels univariater und multivariater logistischer Regression sowie Cox-Regressionsmodellen bewertet und adjustiert für SAPS3 („simplified acute physiology score 3“), Laktatspiegel, Alter, Geschlecht und Komorbiditäten. Interaktionsanalysen zur Auswirkung von Kofaktoren auf die Mortalitätsprognose der RDW wurden durchgeführt.

**Ergebnisse:**

Von 2312 Patient:innen hatten 1687 (73,0 %) eine RDW < 15 und 625 (27,0 %) eine RDW ≥ 15. Patient:innen mit RDW ≥ 15 waren älter (*p* < 0,001), wiesen höhere Werte im SAPS3 (*p* < 0,001) und mehr Komorbiditäten auf. Eine erhöhte RDW war unabhängig mit erhöhter 30-Tage- und Einjahresmortalität assoziiert. In der univariaten Analyse war jede RDW-Einheit mit höherer 30-Tage-Mortalität (Hazard Ratio, HR: 1,169; 95 %-Konfidenzintervall, 95 %-KI: 1,110–1,230; *p* < 0,001) und Einjahresmortalität (HR: 1,153; 95 %-KI: 1,122–1,186; *p* < 0,001) verknüpft. Eine RDW ≥ 15 erhöhte signifikant das Risiko der 30-Tage- (HR: 3,247, 95 %-KI: 2,352–4,482, *p* < 0,001) und Einjahresmortalität (HR: 3,278; 95 %-KI: 2,654–4,048; *p* < 0,001). Interaktionsanalysen zeigten, dass nur Laktatspiegel und präexistierende Lungenerkrankungen die Mortalitätsprognose der RDW beeinflussten. Die RDW trug zur Verbesserung der Modellgüte multivariater Regressionsmodelle bei, wie durch das Akaike-Informationskriterium (AIC) demonstriert wurde.

**Schlussfolgerung:**

Die postoperative RDW ist ein zuverlässiger und kosteneffektiver Marker zur Vorhersage der Kurz- und Langzeitmortalität bei chirurgischen Patient:innen. Ein RDW-Grenzwert von 15 identifiziert Hochrisikopatient:innen und könnte die postoperative Risikostratifizierung verbessern bzw. Patient:innen identifizieren, die von intensiviertem postoperativem Follow-up profitieren könnten.

## Introduction

The red cell distribution width (RDW) quantifies the variability in erythrocyte size. It is calculated from the mean corpuscular volume (MCV) and its standard deviation as part of a complete blood count. Elevated RDW has been linked to increased mortality across various conditions, including cardiovascular disease [1], chronic kidney disease [2], liver disease [3], cancer [4], and chronic pulmonary disease [5]. In critically ill patients, particularly those with sepsis, RDW has emerged as a prognostic biomarker for adverse outcomes [6]. Despite its widespread availability and ease of measurement, the precise pathophysiological mechanisms underlying RDW elevation remain incompletely understood. Inflammation, oxidative stress, impaired erythropoiesis, ageing, and nutritional deficiencies have been implicated as contributing factors, potentially by affecting the half-life of the red blood cells in the circulation [7, 8]. The cause appears to be the delayed elimination of erythrocytes from the blood. Normally, erythrocytes are removed from circulation after 100–120 days, during which they gradually lose volume.

A compensatory response to various pathological conditions, particularly inflammation, leads to delayed erythrocyte elimination and, consequently, to an increased RDW [9]. The thereby increased RDW reflects an unspecific response of the body to various unphysiological conditions that leads to preservation of the red cell mass.

The use of RDW for prognosis estimation is not only possible in humans but also in dogs. Ludwik et al. [10] were able to show that increased RDW is also associated with a poorer prognosis in hospitalized dogs. Interestingly, this did not apply to cats, where the slightly different erythropoiesis is a possible explanation.

The role of RDW in surgical patients has garnered increasing interest. Elevated preoperative RDW has been associated with increased perioperative risk [11], and dynamic changes in RDW following cardiac surgery have been linked to early postoperative complications [12]. While cardiac surgery patients have been extensively studied, the prognostic value of RDW in surgical patients remains largely unexplored, representing an important knowledge gap in perioperative medicine. Data on postoperative RDW as a predictor of both short- and long-term mortality in this population remain scarce. Identifying a simple and reliable biomarker for postoperative risk stratification is of particular clinical relevance, as early identification of high-risk patients could facilitate targeted follow-up strategies, potentially improving long-term outcomes.

In our previous work, we demonstrated that an RDW threshold of 15 is independently associated with increased mortality in intensive care unit (ICU) sepsis patients, irrespective of the analytical platform used [6]. Given the known inter-device variability in RDW measurement and calculation, the use of a standardized cutoff appears critical. Our earlier findings suggest that an RDW cutoff of 15 serves as a robust and device-independent threshold, as evidenced by a cohort using various different devices for measuring RDW. To ensure the broader applicability of our current findings across different cohorts and analytical platforms, we maintained this threshold in the present analysis. Based on these prior results and the established prognostic relevance of RDW in a range of critical illnesses, we hypothesize that postoperative RDW, particularly when exceeding a value of 15, may serve as a valuable predictor of mortality in patients undergoing surgery.

This study aims to evaluate whether a single postoperative RDW measurement provides prognostic information regarding 30-day and 1‑year mortality in surgical patients admitted to intensive care. Additionally, we assess whether an RDW threshold of 15 can reliably stratify postoperative risk and whether RDW retains independent prognostic value after adjusting for established risk factors such as the simplified acute physiology score 3 (SAPS3), lactate levels, and preexisting comorbidities. The clinical implications of this research extend beyond risk stratification, potentially enabling the development of targeted post-discharge monitoring protocols and interventions for high-risk patients, thus improving resource allocation and ultimately enhancing patient outcomes through personalized postoperative care pathways.

## Methods

### Study population and ethics

This retrospective cohort study aimed to evaluate whether postoperative RDW serves as an independent predictor of short- and long-term mortality in surgical ICU patients.

We analyzed patients admitted to a 14-bed mixed surgical ICU at a university hospital over a 2-year period. Patients up to 80 years of age were included to ensure comparability with our previous work, from which we also adopted the RDW cutoff value [6]. The primary outcomes were 30-day and 12-month mortality. The RDW was assessed as both a continuous variable and as a dichotomized variable (RDW ≥ 15 vs. RDW < 15). Laboratory parameters, including RDW, were measured within 2 h postoperatively. Sensitivity analyses were conducted for SAPS3, age, sex, lactate levels, pre-existing diabetes, arterial hypertension, renal insufficiency, lung disease, and red blood cell (RBC) transfusions received during the hospital stay.

Data were extracted from the Salzburg Intensive Care Database (SICdb) [13], which integrates patient information from the ICU data management system (iMDsoft MetaVision ICU) and the hospital’s electronic health record (ORBIS). The SICdb includes admission, discharge, procedural, and ICD-10-coded data as well as medication records and interventions (e.g., renal replacement therapy and mechanical ventilation).

All data were fully pseudonymized according to the European General Data Protection Regulation (GDPR) [14] and de-identified following HIPAA Privacy Rule standards [15]. The study adhered to the RECORD guidelines for observational studies using routine healthcare data [16]. Ethical approval was obtained from the State Ethics Commission of Salzburg, Austria (EK Nr: 1115/2021). Given the use of de-identified data, written informed consent was waived.

### Statistical analysis

Continuous variables are reported as median (interquartile range, IQR) and compared using the Mann–Whitney U test or as mean ± standard deviation (SD) and compared using the Student’s *t*-test, depending on the data distribution. Categorical variables were analyzed using the chi-square test or Fisher’s exact test, as appropriate.

To assess the association between RDW and 30-day mortality, we performed univariable and multivariable logistic regression analyses. For assessment of mortality during the 1‑year period following ICU admission, we performed cox regression analyses. To adjust for potential confounding factors, a multivariable regression analysis was conducted in an adjusted model, accounting for SAPS3, gender, maximum lactate level, prior red cell transfusions, first hemoglobin after surgery, first C‑reactive protein (CRP) level after surgery, norepinephrine dose, and site of surgery.

To investigate the impact of various comorbidities and other cofactors on the mortality-predictive value of RDW, interaction analyses were performed with RDW as a continuous variable and the following parameters: transfused RBCs, maximum lactate level, chronic renal insufficiency, chronic lung disease, arterial hypertension, diabetes, gender, first postoperative creatinine, first postoperative MCV, SAPS3, age at admission, and site of surgery.

Furthermore, for exploratory purposes, a univariable analysis was conducted to evaluate the predictive value of RDW for acute kidney injury on day 3 using multinominal logistic regression.

We report odds ratios (ORs) and hazard ratios (HRs) with 95% confidence intervals (CIs). All statistical tests were two sided, and a *p*-value of < 0.05 was considered statistically significant. Model discrimination was assessed using Nagelkerke’s R^2^ and the Akaike information criterion (AIC) including the log-likelihood. Statistical analyses were performed using Stata/IC 17 (StataCorp LLC, College Station, TX, USA).

## Results

### Study population and characteristics

This retrospective observational study included 2312 patients who underwent surgery and were subsequently admitted to the intensive care unit (ICU). Based on a predefined threshold, 1687 patients (73.0%) had RDW < 15, while 625 patients (27.0%) had RDW ≥ 15. Patients with elevated RDW were significantly older (*p* < 0.001) and had higher SAPS3 (*p* < 0.001), indicating greater illness severity. C‑reactive protein levels were also significantly higher in the elevated RDW group (*p* < 0.001), suggesting more pronounced inflammatory responses. The prevalence of comorbidities, including diabetes mellitus, chronic lung disease, and renal insufficiency, was significantly higher in patients with RDW ≥ 15 (all *p* < 0.05). Additionally, patients with elevated RDW were more likely to have received red blood cell transfusions prior to ICU admission (*p* < 0.001). However, lactate and creatinine levels did not differ significantly between the two groups. Detailed demographic and clinical characteristics are presented in Table 1, and the distribution of surgical sites is summarized in Table 2. All laboratory values were obtained within 2 h of ICU admission to ensure temporal consistency. The variance inflation factor (VIF) was calculated for all variables in the model. The results consistently showed VIF values below 5, indicating that multicollinearity was not a concern in our analysis. Therefore, there was no need to exclude any variables due to collinearity issues.

**Table 1:** Patient characteristics according to red cell distribution width (RDW) level

Patient characteristic	Total	RDW < 15	RDW ≥ 15	*p*-value
	*N* = 2312	*N* = 1687	*N* = 625	
*Age at admission (years)*	70 (60–80)	65 (55–75)	70 (65–80)	< 0.001
*Age ≥* *70 years (0* *=* *no, 1* *=* *yes)*	41% (942)	37% (631)	50% (311)	< 0.001
*Male sex (0* *=* *female, 1* *=* *male)*	63% (1457)	66% (1118)	54% (339)	< 0.001
*SAPS3*	42 (34–51)	41 (33–49)	47 (38–57)	< 0.001
*SAPS3 ≥* *43 (0* *=* *no, 1* *=* *yes)*	49% (1130)	44% (744)	62% (386)	< 0.001
*KDIGO AKI at 168 h (0* *=* *no AKI, 1* *=* *AKI)*				< 0.001
No AKI	46% (1053)	48% (817)	38% (236)	
Stage 1 AKI	20% (466)	20% (341)	20% (125)	
Stage 2 AKI	29% (659)	27% (459)	32% (200)	
Stage 3 AKI	6% (134)	4% (70)	10% (64)	
*Length of ICU stay (hours)*	32 (23–75)	32 (23–73)	30 (23–92)	0.30
*30-day mortality (0* *=* *survivor, 1* *=* *deceased)*	7% (163)	5% (78)	14% (85)	< 0.001
*1‑year mortality (0* *=* *survivor, 1* *=* *deceased)*	15% (351)	10% (170)	29% (181)	< 0.001
*Time to 30-day mortality (days)*	30 (30–30)	30 (30–30)	30 (30–30)	< 0.001
*Time to 1‑year mortality (days)*	365 (365–365)	365 (365–365)	365 (136–365)	< 0.001
*Max hemoglobin first week (g/dL)*	11.4 (9.9–12.9)	11.9 (10.4–13.3)	10.1 (9.1–11.3)	< 0.001
*Min hemoglobin first week (g/dL)*	9.1 (7.4–11.1)	9.7 (7.7–11.6)	7.9 (7.0–9.4)	< 0.001
*Max. thrombocytes first week (G/L)*	258 (202–332)	251 (201–313)	282 (204–371)	< 0.001
*Min. thrombocytes first week (G/L)*	182 (140–237)	178 (140–227)	196 (139–272)	< 0.001
*Max. leukocytes first week (G/L)*	12.2 (9.4–16.1)	12.0 (9.4–15.7)	12.8 (9.4–16.9)	0.017
*Min. leukocytes first week (G/L)*	7.3 (5.8–9.3)	7.3 (5.9–9.2)	7.4 (5.6–9.6)	0.88
*Max. lactate first 24* *h (mmol/L)*	1.8 (1.3–2.5)	1.8 (1.3–2.5)	1.8 (1.3–2.5)	0.27
*Max. lactate first week (mmol/L)*	1.9 (1.4–2.8)	1.9 (1.3–2.7)	1.9 (1.4–2.8)	0.019
*Lactate on day 1 (mmol/L)*	1.8 (1.3–2.5)	1.8 (1.3–2.5)	1.8 (1.3–2.5)	0.32
*Lactate ≥* *2* *mmol/L (0* *=* *no, 1* *=* *yes)*	37% (856)	37% (614)	39% (242)	0.37
*RDW count*	13.9 (13.0–15.3)	13.4 (12.8–14.1)	16.6 (15.6–18.1)	< 0.001
*RDW on ICU admission*	13.8 (12.9–15.1)	13.2 (12.7–13.9)	16.4 (15.6–17.8)	< 0.001
*RDW on day 1*	14.0 (13.2–15.5)	13.5 (12.9–14.3)	16.7 (15.8–18.0)	< 0.001
*RDW on day 2*	14.4 (13.3–15.9)	13.7 (13.0–14.6)	16.8 (15.9–18.4)	< 0.001
*Mean RDW over 72* *h*	13.9 (13.1–15.3)	13.4 (12.8–14.1)	16.6 (15.6–18.2)	< 0.001
*First RDW measurement*	13.8 (12.9–15.1)	13.2 (12.7–13.9)	16.4 (15.6–17.8)	< 0.001
*First creatinine measurement (mg/dL)*	0.9 (0.7–1.1)	0.9 (0.7–1.1)	0.9 (0.7–1.4)	0.002
*Max. creatinine first week (mg/dL)*	1.0 (0.8–1.4)	1.0 (0.8–1.2)	1.1 (0.8–1.8)	< 0.001
*First international normalized ratio*	1.20 (1.20–1.30)	1.20 (1.20–1.30)	1.20 (1.20–1.40)	< 0.001
*First mean corpuscular hemoglobin (pg)*	30.3 (29.0–31.6)	30.6 (29.5–31.8)	29.1 (26.8–30.8)	< 0.001
*First mean corpuscular volume (fL)*	89 (86-92)	89 (86-92)	87 (82-92)	< 0.001
*First mean corpuscular hemoglobin concentration (g/dL)*	34.0 (33.2–34.8)	34.3 (33.5–35.0)	33.2 (32.0–34.1)	< 0.001
*First C‑reactive protein (mg/L)*	1.1 (0.2–5.1)	0.8 (0.2–4.0)	2.8 (0.6–9.2)	< 0.001
*Urine output first 24* *h (mL)*	1425 (915-2150)	1505 (995-2290)	1240 (775-1840)	< 0.001
*Transfusion category (defined in detail)*				< 0.001
No transfusion	79% (1830)	84% (1409)	67% (421)	
One transfusion	7% (171)	6% (98)	12% (73)	
Two transfusions	6% (138)	5% (83)	9% (55)	
> 2 transfusions	7% (173)	6% (97)	12% (76)	
*Received transfusion (0* *=* *no, 1* *=* *yes)*	21% (482)	16% (278)	33% (204)	< 0.001
*Norepinephrine dose (µg/kg/min, avg. first week)*	0.053 (0.031–0.087)	0.050 (0.030–0.084)	0.060 (0.037–0.096)	0.002
*Diabetes (0* *=* *no, 1* *=* *yes)*	20% (472)	18% (296)	28% (176)	< 0.001
*Arterial hypertension (0* *=* *no, 1* *=* *yes)*	53% (1224)	52% (870)	57% (354)	0.030
*Chronic lung disease (0* *=* *no, 1* *=* *yes)*	13% (290)	11% (182)	17% (108)	< 0.001
*Chronic kidney disease*	15% (350)	12% (195)	25% (155)	< 0.001
*Duration of mechanical ventilation (h)*	7 (3-14)	7 (3-14)	6 (3-16)	0.16

*AKI* Acute kidney disease, *CI* confidence interval, *CRP* C-reactive protein, *KDIGO* Kidney Disease: Improving Global Outcomes, *SAPS3* Simplified Acute Physiology Score 3, *RDW* red cell distribution width

**Table 2:** Site of surgery according to red cell distribution width (RDW) level

Site of surgery	Total	RDW < 15	RDW ≥ 15	*P*-value
Abdomen	16% (370)	14% (238)	21% (132)	< 0.001
Vascular	10% (240)	10% (172)	11% (68)	0.63
Cardiac	12% (284)	15% (251)	5% (33)	< 0.001
Neurosurgery	1% (23)	1% (17)	1% (6)	0.92
Thorax	2% (49)	2% (32)	3% (17)	0.22
Trauma	4% (94)	4% (69)	4% (25)	0.92
Obstetrics & gynecology	2% (39)	2% (26)	2% (13)	0.37
Other	20% (455)	20% (335)	19% (120)	0.72
Unknown	33% (758)	32% (547)	34% (211)	0.54

This table presents the distribution of surgical sites among patients stratified by RDW (RDW < 15 vs. ≥ 15). Values are shown as percentages and absolute numbers. Comparisons between groups were performed using the chi-square (χ^2^) test to assess whether the distribution of surgical sites differed significantly between RDW groups. A *p*-value < 0.05 was considered statistically significant. *RDW* red cell distribution width

### RDW as a continuous predictor of mortality

#### 30-day mortality

**Fig. 1:**
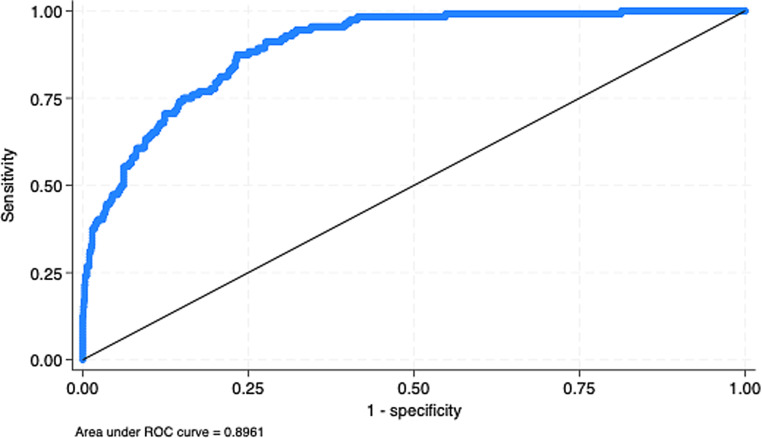
Kaplan–Meier survival curve comparing survival probabilities over 365 days

In univariable analysis, RDW demonstrated significant prognostic value when assessed as a continuous variable. Each unit increase in RDW was associated with an elevated risk of 30-day mortality (HR 1.169, 95% CI 1.110–1.230; *p* < 0.001). The independent association between RDW and 30-day mortality persisted in the adjusted model (see Methods; adjusted HR 1.097, 95% CI 1.004–1.198; *p* = 0.041; R2 0.420, VIF 1.33, AUROC 0.896; Fig. 1).

#### 1-year mortality

The RDW also showed significant prognostic value for 1‑year mortality in univariable analysis when assessed as a continuous variable. Each unit increase in RDW was associated with an elevated risk of 1‑year mortality (HR 1.153, 95% CI 1.122–1.186; *p* < 0.001). The independent association between RDW and 1‑year mortality remained significant in the adjusted model (adjusted HR 1.108, 95% CI 1.061–1.158; *p* < 0.001).

### RDW as a binary predictor of mortality

When RDW was analyzed as a binary variable with a threshold of 15, elevated RDW demonstrated even stronger associations with adverse outcomes.

#### 30-day mortality

In univariable analysis, RDW ≥ 15 was significantly associated with an elevated risk of 30-day mortality (HR 3.247, 95% CI 2.352–4.482; *p* < 0.001). After using the adjusted model, the independent association between RDW ≥ 15 and 30-day mortality remained significant (adjusted HR 2.287, 95% CI 1.388–3.766; *p* < 0.001; R2 0.43, VIF 1.33, AUROC 0.898; sensitivity 0.53, specificity 0.75).

#### 1-year mortality

In the univariable analysis, RDW ≥ 15 again demonstrated significant prognostic value for 1‑year mortality (HR 3.278, 95% CI 2.654–4.048; *p* < 0.001). After using the adjusted model, the independent association between RDW and 1‑year mortality persisted (adjusted HR 2.184, 95% CI 1.632–2.921; *p* < 0.001; AUROC 0.63; sensitivity 0.51, specificity 0.75).

### Interaction terms between RDW and covariates

#### 30-day mortality

Interaction terms between RDW as a continuous variable and covariates for 30-day mortality were calculated (Table 3). Only maximum lactate level and chronic lung disease significantly influenced the predictive value of RDW for 30-day mortality.

**Table 3:** Interaction between covariates and RDWc affecting 30-day mortality

Interaction	Odds ratio	95% CI	*p*-value
Transfused RBCs and RDWc	0.91	0.81–1.02	0.102
Max. lactate level and RDWc	0.97	0.95–0.99	0.027
Chronic renal insufficiency and RDWc	0.91	0.81–1.03	0.138
Chronic lung disease and RDWc	0.86	0.74–0.99	0.038
Arterial hypertension and RDWc	0.94	0.85–1.04	0.236
Diabetes and RDWc	0.94	0.83–1.06	0.315
Male gender and RDWc	0.98	0.88–1.08	0.655
First postoperative creatinine and RDWc	1.05	1.00–1.11	0.055
First postoperative MCV and RDWc	1.01	0.99–1.01	0.095
First postoperative CRP	1.00	0.99–1.00	0.409
First postoperative hemoglobin	1.02	0.99–1.05	0.197
SAPS3 and RDWc	0.99	0.99–1.00	0.079
Age at admission and RDWc	1	0.99–1.00	0.792
Vascular surgery group and RDWc	1.05	0.88–1.25	0.617
Cardiac surgery group and RDWc	1.08	0.82–1.43	0.587
Thorax surgery group and RDWc	1.11	0.77–1.59	0.574
Trauma surgery group and RDWc	1.05	0.81–1.35	0.733
Obstetrics and gynecology surgery group and RDWc	1	0.62–1.63	0.996
Non-specified surgery group and RDWc	1.05	0.88–1.24	0.605
Unknown surgery site group and RDWc	1.14	0.98–1.32	0.083

*RDW* red cell distribution width, *SAPS3* Simplified Acute Physiology Score 3

#### 1-year mortality

Interaction terms between RDW as a continuous variable and various covariates for 1‑year mortality were also assessed. Several covariates showed a significant interaction with RDW’s predictive value (Table 4).

**Table 4:** Interaction between covariates and RDWc affecting 1‑year mortality

Interaction	Hazard ratio	95% CI	*p*-value
Transfused RBCs and RDWc	0.95	0.89–1.01	0.093
Max. lactate level and RDWc	0.98	0.97–0.99	0.001
Chronic renal insufficiency and RDWc	0.93	0.86–0.99	0.041
Chronic lung disease and RDWc	0.88	0.81–0.96	0.003
Arterial hypertension and RDWc	0.95	0.89–1.00	0.053
Diabetes and RDWc	0.96	0.90–1.03	0.291
Male gender and RDWc	1.01	0.96–1.07	0.687
First postoperative creatinine and RDWc	1.04	1.01–1.07	0.017
First postoperative MCV and RDWc	1.01	1.00–1.01	< 0.001
First postoperative CRP	1.00	0.99–1.00	0.88
First postoperative hemoglobin	1.02	1.01–1.04	0.001
SAPS3 and RDWc	1	0.99–1.00	0.137
Age at admission and RDWc	1	0.99–1.00	0.045
Vascular surgery group and RDWc	0.99	0.87–1.13	0.899
Cardiac surgery group and RDWc	1.22	1.03–1.43	0.02
Thorax surgery group and RDWc	1.12	0.92–1.37	0.251
Trauma surgery group and RDWc	1.05	0.92–1.20	0.437
Obstetrics and gynecology surgery group and RDWc	0.83	0.52–1.31	0.424
Non-specified surgery group and RDWc	1.08	0.99–1.18	0.093
Unknown surgery site group and RDWc	1.11	1.02–1.21	0.014

*RDW* red cell distribution width, *SAPS3* Simplified Acute Physiology Score 3

### Akaike information criterion calculation

To assess the additional value of RDW, both as a continuous variable and with a threshold of 15, in a multivariable model for predicting 30-day and 1‑year mortality, Akaike information criterion (AIC) calculations were performed. The AIC was applied to the multivariable model including SAPS3, gender, maximum lactate level, prior red cell transfusions, and site of surgery, once with RDW as a continuous variable and once with RDW ≥ 15 as a binary variable.

#### AIC for 30-day mortality with RDW as a continuous variable

The multivariable model including RDW as a continuous variable (RDWc) yielded an AIC of 917.559 with a log-likelihood ratio of −445.75. After excluding RDW from the model, the AIC increased to 922.134 with a log-likelihood ratio for the model of −449.067, indicating that including RDW improved the prediction of 30-day mortality.

#### AIC for 30-day mortality with RDW as a binary variable

The model including RDW ≥ 15 had an AIC of 909.3584 with a log-likelihood of −441.6792. After excluding RDW ≥ 15, the AIC increased to 922.1347 with a log likelihood ratio of −449.0674, further suggesting that RDW ≥ 15 contributes to improving 30-day mortality prediction.

#### AIC for 1-year mortality with RDW as a continuous variable

In the multivariable Cox regression model, the inclusion of RDW as a continuous variable resulted in an AIC of 4952.413 with a log-likelihood of −2463.206. After removing RDW, the AIC increased to 4980.72 with a log-likelihood for the model of −2478.36, confirming that RDW also enhances 1‑year mortality prediction.

#### AIC for 1-year mortality with RDW as a binary variable

For 1‑year mortality, the model including RDW ≥ 15 had an AIC of 4929.847 and a log-likelihood of −2451.924. Excluding RDW ≥ 15 from the model increased the AIC to 4980.72 with a log-likelihood for the model of −2478.36, highlighting the contribution of RDW ≥ 15 to predicting 1‑year mortality.

### RDW as a predictor of Acute Kidney Injury after a 3-day ICU stay

In an univariable exploratory analysis, RDW as a continuous variable showed significant prognostic value for the development of AKI (Acute Kidney Injury) after a 3-day ICU stay. Each unit increase in RDW was associated with an elevated risk of stage 3 AKI (RR 1.20, 95% CI 1.13–1.28; *p* < 0.001). RDW ≥ 15 was also significantly associated with a higher risk of stage 2 (RR 1.51, 95% CI 1.21–1.88; *p* < 0.001) and stage 3 AKI (RR 3.17, 95% CI 2.19–4.58; *p* < 0.001). However, for stage 1 AKI, the association was not significant (RR 1.27, 95% CI 0.99–1.63; *p* = 0.063).

## Discussion

In addition to the immediate success of surgery, the medium- and long-term postoperative outcomes are of crucial importance for patients. Especially after major surgeries that necessitate intensive care unit (ICU) admission, accurate risk assessment can be critical for the medium- and long-term prognosis. Patients with higher postoperative complication and mortality risks may benefit from more intensive follow-up care or referral to appropriate post-discharge care structures. It has been demonstrated, for example by Fowler et al., that postoperative complications increase the long-term mortality risk. However, due to limited resources and the demographic changes in society, which lead to a higher number of ICU admissions, not all postoperative patients can be integrated into these intensive follow-up systems. Therefore, it is essential to identify patients at a high risk of postoperative complications or those with an elevated postoperative mortality risk.

The use of severity scores, such as SAPS3, or the assessment of relevant comorbidities like the ASA (American Society of Anesthesiologists) classification, is often complicated and prone to subjective misjudgments.

In our study of 2312 surgical patients who were admitted to the ICU postoperatively, we demonstrated that the postoperative determination of a single RDW measurement has significant predictive value for the mortality risk at 30 days and 1 year.

The association between RDW and mortality risk was evident both when RDW was used as a continuous variable, where increasing RDW values were linked to higher mortality, as well as when using a threshold of RDW ≥ 15. This threshold was chosen after we showed in an earlier study that RDW ≥ 15 was associated with increased mortality in ICU sepsis patients [6].

**Fig. 2:**
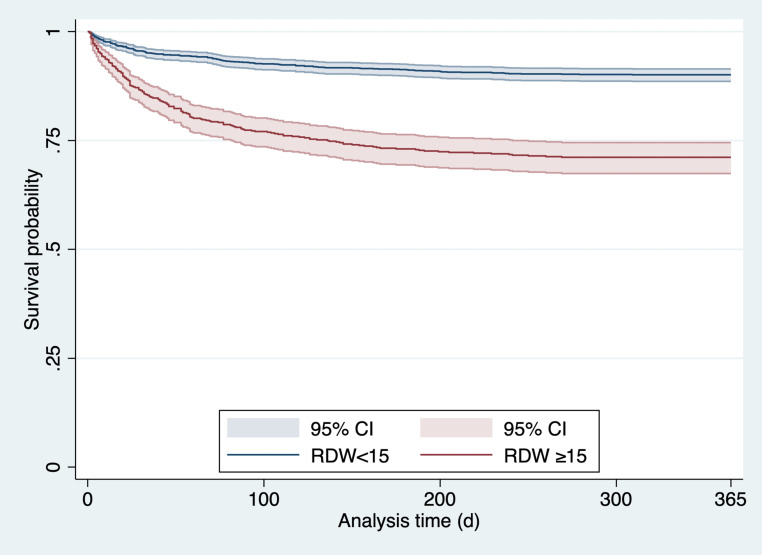
Receiver operating characteristic curve for red cell distribution width (*RDW*) as a continuous variable. *CI *confidence interval

Overall, both when RDW was treated as a continuous variable and when using the RDW ≥ 15 threshold, there was a significant increase in the 30-day (HR 1.169, 95% CI 1.110–1.230; *p* < 0.001) and 1‑year (HR 1.153, 95% CI 1.122–1.186; *p* < 0.001) mortality risks. Using the RDW ≥ 15 threshold showed an even stronger association with mortality risk (30-day mortality HR 3.247, 95% CI 2.352–4.482; *p* < 0.001; 1‑year mortality HR 3.278, 95% CI 2.654–4.048; *p* < 0.001; Fig. 2).

Various factors can influence RDW. For instance, Lippi et al. [7] demonstrated that RDW varies depending on age and sex, while Spadaro et al. [18] described RDW changes based on erythrocyte transfusions. To assess the influence of various cofactors on RDW as a mortality risk predictor, we conducted interaction analyses with covariates and RDW as a continuous variable (Tables 3 and 4). For 30-day mortality, only lactate levels and chronic lung disease had a significant impact on RDW. Both covariates reduced the odds ratio of RDW for 30-day mortality. The significant negative interaction with maximum lactate can be explained by lactate levels serving as a marker for the acute severity of disease, which may have higher prognostic value than RDW. Taking into account the dependence of RDW on erythropoiesis and erythrocyte lifespan, which are much slower processes than, for example, lactate generation, RDW can be considered as more of a medium- to long-term prognostic marker. The same applies to chronic lung disease, where long-standing hematologic adaptations such as secondary erythrocytosis or chronic hypoxemia may already be captured by RDW, reducing its additive predictive value. However, it cannot be ruled out that the described interactions with these two factors are incidental, as these analyses were purely descriptive.

In the 1‑year mortality interaction analysis, several covariates showed significant interactions. Lactate and chronic lung disease similarly reduced RDW’s association with mortality, as seen in the 30-day mortality interaction analysis. A similar result was found with chronic renal insufficiency, and there was a borderline significant interaction with age at admission. It again appears plausible that renal insufficiency, as a highly relevant prognostic factor, has a greater influence on 1‑year mortality than RDW.

In the comprehensive interaction analysis, including the site of surgery, no significant effect on RDW as a predictive factor for 30-day or 1‑year mortality was found (RDWc 1‑year mortality HR 1.09; *p* = 0.011; RDWc 30-day mortality HR 1.10; *p* = 0.077). This suggests that postoperative RDW is a reliable predictor of 30-day and 1‑year mortality in surgical patients, regardless of comorbidities or the type of surgery.

To clarify whether postoperative RDW can further improve the risk assessment of 30-day and 1‑year mortality in a multivariable mortality-prediction model (including SAPS3, gender, maximum lactate level, prior red cell transfusions, and site of surgery), AIC calculations were performed. The results consistently showed an improvement in the mortality-prediction model for both 30-day and 1‑year mortality when RDW as a continuous variable or RDW ≥ 15 was incorporated into the model.

Previous data have shown that there is an independent association between the RDW/albumin ratio and AKI in intensive care patients with sepsis [19]. Therefore, we performed an exploratory analysis assessing the association of RDW and AKI in our patient cohort and were able to demonstrate that RDW was significantly associated with a higher risk of developing AKI by day 3 post-ICU admission. Although there are no specific therapies or prophylactic options to prevent AKI, identifying patients at an increased risk of AKI can still help to reduce the frequency of AKI, at least by avoiding nephrotoxic medications or optimizing renal hemodynamics. In this context, RDW may serve as a useful marker for identifying patients at risk of AKI, and further research into its potential role as a predictor of AKI would be of considerable interest.

These findings suggest that RDW, particularly with a cutoff of 15, is a valuable tool for predicting both short- and long-term postoperative outcomes in surgical patients, especially for identifying individuals at a higher risk of mortality. In this context, a single postoperative RDW measurement appears to serve as a marker of overall disease severity, improving the prognostic assessment of these patients. Furthermore, most other prognostically relevant factors, such as SAPS3 or age, did not show any negative interaction with the prognostic value of RDW, thereby underscoring the robust value of RDW as a prognostic factor. This may reflect the complex pathophysiology underlying RDW changes, potentially representing the overall health status of the body.

### Strengths and limitations

#### Strengths

This study analyses a relatively large and heterogeneous cohort of postoperative patients. As all patients were treated in a single ICU under uniform clinical standards, variability in the quality of care as a potential confounder was minimized. The availability of 1‑year mortality data further enables an assessment of RDW in relation to long-term prognosis. Moreover, our prior work in a large and diverse patient population allowed for the use of an analyzer-independent RDW cutoff, thereby enhancing the generalizability of the findings.

#### Limitations

This retrospective study is subject to potential biases, including unmeasured confounders such as inflammatory status, nutritional deficits, or functional impairment, which may affect both RDW and mortality. Additionally, reverse causation cannot be excluded, as elevated RDW may primarily reflect the underlying disease severity. The inclusion of only ICU-admitted postoperative patients may also introduce selection bias and limit generalizability to broader surgical populations.

To strengthen these findings, prospective studies should be conducted, ideally including more comprehensive clinical data and also covering surgical patients not requiring ICU admittance. Measuring RDW at multiple timepoints and integrating it into prospective risk models could further help to clarify its independent prognostic role and clinical utility in postoperative care.

## Conclusion

A single RDW measurement obtained immediately postoperatively may represent a cost-effective and readily accessible tool for early risk stratification. In the short-term perioperative setting, particularly under resource-limited conditions, RDW could provide a pragmatic alternative to more complex scoring systems such as SAPS3, which rely on a broader range of laboratory parameters, and may serve as a valuable complementary marker to refine individual risk stratification and support early, resource-efficient prognostic assessment in critically ill patients. In the longer term, RDW may contribute to the development of individualized follow-up strategies for high-risk patients or, at a minimum, help identify those who warrant more comprehensive postoperative risk assessment.
